# What is uric acid concentration in urine in patients with uric acid kidney stones? - a case study

**DOI:** 10.11613/BM.2025.031001

**Published:** 2025-08-15

**Authors:** Tomáš Šálek, Pavel Musil, Irena Zlatníková

**Affiliations:** 1 Department of Clinical Biochemistry and Pharmacology, Tomas Bata Hospital in Zlín, Zlín, Czech Republic; 2 Department of Health Care Sciences, Faculty of Humanities, Tomas Bata University in Zlín, Zlín, Czech Republic

**Keywords:** nephrolithiasis, uric acid, urine specimen collection, urine, specimen preservation

## Abstract

This case report describes a patient with uric acid kidney stones. Alkalization therapy using mainly potassium citrate is the first-choice treatment. When hyperuricosuria > 4 mmol/24 hours is present, xanthine oxidase inhibitors are added. It implies that accurate urine uric acid measurement is of high importance. Uric acid was measured in a 24-hour collection and a second-morning sample. Urine uric acid was measured after sample alkalization to pH > 6.5 and heating to 56 °C for 10 minutes, and for educational reasons without sample treatment. The uric acid excretion in the sample without alkalization in the 24-hour collection was 2.436 mmol, after alkalization, the excretion was 4.650 mmol/24 hours. Sample alkalization led to a prescription for xanthine oxidase inhibitor medication that is indicated as the second-line therapy when hyperuricosuria > 4 mmol/24 hours is present. This case study shows how the correct preanalytical phase is essential for medical decision-making.

## Graphical abstract

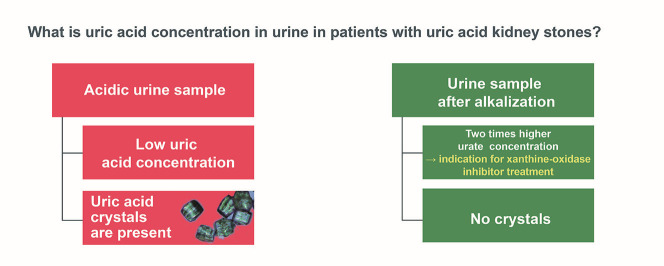

## Highlights

• Uric acid crystals are formed in overly acidic urine

• Sample alkalization is needed for urine uric acid measurement

• 24-hour collected urine or second-morning spot sample could have been used for treatment decisions in this patient

## Introduction

Kidney stone disease is highly prevalent worldwide, with the prevalence in North America at 7-13%. The prevalence in Europe is 5-9% (*1*). The first steps in diagnosis include evaluation of detailed medical and dietary history, kidney stone analysis, and measurement of urine and serum kidney stone risk factors. At least one 24-hour urine collection is recommended to identify the risk factors. Therapy aims to avoid recurrent disease by decreasing urine saturation and concentrations of lithogenic substances and increasing levels of kidney stone inhibitors (*2*).

Uric acid nephrolithiasis is a systemic metabolic disorder and the third most common type of kidney stone in the industrialized world, accounting for about 10% of all stone formers (*3*). The uric acid dissociation constant (pKa) is 5.35 at 37 °C. The overly acidic urine promotes the protonation of water-soluble urate to the poorly soluble uric acid (*4*).

The local Ethics Committee approved the publication of case study No. 2024/18. The patient signed the informed consent regarding the publication of his case study.

This case study presents a patient with a uric acid kidney stone with falsely low urine uric concentration when measured without alkalization due to crystal formation in an acidic environment.

A 55-year-old male has suffered from kidney stones since March 2023. The infrared spectroscopy revealed a 100% uric acid kidney stone composition. In November 2024, the next stone passed with the same composition. In January 2025, he was referred to the Metabolic Clinic for kidney stone prevention. His medical history included obesity, hypercholesterolemia, hypertension, type 2 diabetes mellitus, and reflux esophagitis. His medication included atorvastatin 20 mg, perindopril 5 mg, bisoprolol 5 mg, metformin 1700 mg, and esomeprazole 40mg daily. Ha had no allergy. The physical examination showed a blood pressure of 131/95 mmHg, a height of 187 cm, and a body weight of 127 kg (body mass index of 36 kg/m^2^). Metabolic evaluation of serum and urine kidney stone risk factors was performed.

## Laboratory analyses

The results of basic serum chemistry tests are shown in Table 1. Urine kidney stone risk factors were analyzed in the 24-hour collection sample and spot second-morning urine. Results from both urine samples are shown in Table 2. In addition, for educational reasons, uric acid concentrations were measured before alkalization, and the results are shown in a separate row.

**Table 1:** Serum laboratory test results (selected methods)

**Serum laboratory test (unit)**	**Result**	**Reference range**
Sodium (mmol/L)	140	136-144
Potassium (mmol/L)	4.3	3.8-5.1
Chloride (mmol/L)	108	95-107
Total calcium (mmol/L)	2.21	2.10-2.55
Magnesium (mmol/L)	0.83	0.80-0.94
Inorganic phosphate (mmol/L)	0.99	0.81-1.45
Urea (mmol/L)	4.8	3.0-8.0
Creatinine (µmol/L)	87	64-104
eGFR (CKD-EPI equation) from serum creatinine (mL/min/1.73m^2^)	86	90-150
Uric acid (µmol/L)	366	140-360
Serum HCO_3_^-^ (mmol/L)	19.8	22.0-28.0
eGFR - estimated glomerular filtration rate. CKD-EPI - Chronic Kidney Disease Epidemiology Collaboration.		

**Table 2:** Urine laboratory test results (selected methods)

**Urine laboratory test (unit)**	**24 hour collected urine result**	**Second-morning spot sample result**	**Reference range**
Volume (L)	1.2	–	1.5-3.0
pH	4.96	4.84	–
Uric acid before alkalization (mmol)	2.436	–	–
Uric acid to creatinine ratio, alkalized test	–	0.31	0.10-0.30
Uric acid, alkalized test (mmol)	4.650	–	2.0-4.0
Citrate to creatinine ratio	–	0.18	> 0.15
Citrate (mmol)	5.1	–	> 2.5
Sodium (mmol)	178	–	0-87
Calcium (mmol)	5.19	–	2.0-5.0
Magnesium (mmol)	3.9		3.0-5.0
Inorganic phosphate (mmol)	30	–	13-34
Oxalate (mmol)	0.336	–	0.00-0.50
Ammonium (mmol)	34	–	15-50
Sulfate (mmol)	21	–	7-47
Urea (mmol)	401	–	330-580

Urine was collected in a 5-liter polyethylene container with 2.5 g of thymol added in advance at the laboratory (1 g/L urine, diluted from a stock of 10% thymol w/v in isopropanol). The 24-hour urine collection was delivered to the laboratory within two hours after collection, the container was properly mixed, urine volume was measured, and the specimen was divided into the aliquots in 10 mL polypropylene tubes without additives (FL Medical, Padua, Italy). Without further additives, the first 1 mL aliquot was used to immediately measure pH, osmolality, sodium, ammonium, urea, and creatinine. Tthe second 1 mL aliquot was acidified by 10 µL of 6M hydrochloric acid to decrease its pH below two and heated at 56 °C for 10 minutes for calcium, magnesium, and inorganic phosphate measurements. The third 1 mL aliquot was acidified by 20 µL 7M H_3_PO_4_ to measure citrate, oxalate, and sulfate. Before measurement, the sample was heated in a water bath at 56 °C for 30 min to solubilize possible crystals. The fourth 1mL aliquot was alkalized by 10 µL 6M NaOH to pH > 6.5 and heated at 56 °C for 10 minutes for uric acid measurement. This analytical approach (alkalization + heating) was recommended by the Working Group Preanalytical Phase (WG-PRE) and Task and Finish Group Urinalysis (TFG-U) of the European Federation of Clinical Chemistry and Laboratory Medicine (EFLM) (*5*).

When bringing their 24-hour specimen to the laboratory, second-morning urine was collected under a fluid restriction to a maximum of 2 dL before voiding at the laboratory. Thymol was added to the second-morning specimen at the same concentration as in the 24-hour collections of urine. In the laboratory, the same analyses were performed in the spot second-morning urine as in the collected sample.

Calcium, magnesium, inorganic phosphate, uric acid, sodium, potassium, chloride, and creatinine were measured on the Abbott Architect *ci*16200 analyzer (Abbott Laboratories, Abbott Park, USA). The analytical performance characteristics of the urine uric acid method, which is based on the uricase spectrophotometric principle, are: relative bias of 3.70%, intermediate precision of 1.87%, post-alkalization recovery of 96.31%, and linearity range of 0.12-15.42 mmol/L. Oxalate, citrate, and sulfate measurements were carried out by capillary electrophoresis on Lumex CAPEL–205 analyzer (Lumex Instruments Canada, Mission, Canada).

Urine pH was measured by a glass pH electrode using a pH meter (Perphect Meter Model 330, Canton, USA).

## What happened?

The patient had falsely low uric acid concentrations in urine due to acidic pH without sample alkalization.

After urine alkalization, the urine uric acid concentrations markedly increased. Uric acid crystals were formed in acidic urine, leading to lower uric acid concentrations.

## Discussion

This case study shows falsely low urine uric acid concentrations due to the formation of uric acid crystals in acidic urine.

Low urine pH (below 5.5), low urine volume, and hyperuricosuria are key risk factors for uric acid crystal formation. Stone compositional analysis is the definitive diagnostic tool and should be attempted in all patients. Urine pH plays a central role in the diagnosis and monitoring of this type of nephrolithiasis (*6*). Our patient had all these risk factors. Kidney stone analysis was performed by infrared spectroscopy, and his urine pH was measured by pH meter.

Sakhaee *et al.* reported that low urine pH is the most important factor in uric acid kidney stone development. Insulin plays an important role in renal ammonium synthesis. Insulin resistance leads to impaired renal ammonia synthesis and excretion. Decreased urine ammonium excretion in those patients leaves protons in urine unbuffered, leading to lower pH (*7*). Our patient had urine pH even below 5.0 in both the collected and the second-morning samples. The morning sample had a lower pH compared to the 24-hour collected sample. The morning samples are generally more concentrated than the 24-hour collections. It shows that morning urine samples may also be useful for monitoring this type of kidney stone. The reason may also be that 24-hour collections are not popular among patients and are frequently incomplete. In our patient, based on second-morning urine sample results, the patient may also be advised to eat a more plant-based alkaline diet, less acid-producing animal products, and prescribed citrate treatment. Monitoring by imaging techniques like sonography is also needed to evidence the dissolution.

Wiederkehr and Moe reported that urine uric acid concentrations also play a role in uric acid crystal precipitation (*3*). It implies that correct uric acid concentration measurement is important.

Sakhaee also reported that uric acid kidney stones are more frequent in patients with obesity and type 2 diabetes mellitus (*8*). The same clinical features are present in our case study.

The first-line therapy in uric acid kidney stone patients is citrate alkalization to a urine pH of 6.5 (*9*). Citrate alkalization therapy can be prescribed based on uric acid kidney stone composition and acidic urine pH. Xanthine oxidase inhibitors are used in the second step when hyperuricosuria > 4 mmol/24 hours is present (*10*). This case study showed that sample alkalization led to two times higher 24-hour uric acid excretion results. A similar situation was in the spot second-morning sample. It is the reason why urine alkalization is recommended before uric acid measurement.

Many urine constituents change in concentration when the rate of water excretion alters due to variations in fluid intake and diet. Diagnostic decisions should not be based on a single measurement due to this variability, especially in borderlines of diagnostic categories (*11*). All our kidney stone patients are regularly monitored. The more results we have, the better.

## What YOU should /can do in your laboratory to prevent such errors

Alkalization to pH > 6.5 and heating to 56 °C for 10 minutes is needed before urine uric acid measurement.

## Data Availability

All data generated and analyzed in the presented study are included in this published article.

## References

[1] Sorokin I Mamoulakis C Miyazawa K Rodgers A Talati J Lotan Y. Epidemiology of stone disease across the world. World J Urol. 2017;35:1301–20. 10.1007/s00345-017-2008-6 28213860

[2] Kidney Stones: Medical Mangement Guideline - American Urological Association. Available from: https://www.auanet.org/guidelines-and-quality/guidelines/kidney-stones-medical-mangement-guideline. Accessed February 10th 2025.

[3] Wiederkehr MR Moe OW. Uric Acid Nephrolithiasis: A Systemic Metabolic Disorder. Clin Rev Bone Miner Metab. 2011;9:207–17. 10.1007/s12018-011-9106-6 25045326 PMC4100778

[4] Bobulescu IA Park SK Xu LHR Blanco F Poindexter J Adams-Huet B Net Acid Excretion and Urinary Organic Anions in Idiopathic Uric Acid Nephrolithiasis. Clin J Am Soc Nephrol. 2019;14:411–20. 10.2215/CJN.10420818 30745301 PMC6419274

[5] Šálek T Musil P Vermeersch P Marrington R Dikmen ZG Poláchová R Preservation of urine specimens for metabolic evaluation of recurrent urinary stone formers. Clin Chem Lab Med. 2024;63:129–38. 10.1515/cclm-2024-0773 39072394

[6] Shastri S Patel J Sambandam KK Lederer ED. Kidney Stone Pathophysiology, Evaluation and Management: Core Curriculum 2023. Am J Kidney Dis. 2023;82:617–34. 10.1053/j.ajkd.2023.03.017 37565942 PMC11370273

[7] Sakhaee K Adams-Huet B Moe OW Pak CYC. Pathophysiologic basis for normouricosuric uric acid nephrolithiasis. Kidney Int. 2002;62:971–9. 10.1046/j.1523-1755.2002.00508.x 12164880

[8] Sakhaee K. Epidemiology and Clinical Pathophysiology of Uric Acid Kidney Stones. J Nephrol. 2014;27:241–5. 10.1007/s40620-013-0034-z 24497296 PMC4696481

[9] Bhojani N Bjazevic J Wallace B Lee L Kaler KS Dion M Update - 2022 Canadian Urological Association guideline: Evaluation and medical management of the kidney stone patient. Can Urol Assoc J. 2022;16:175–88. 10.5489/cuaj.7872 35623003 PMC9245965

[10] EAU Guidelines on Urolithiasis - Uroweb. Available from: https://uroweb.org/guidelines/urolithiasis/chapter/metabolic-evaluation-and-recurrence-prevention. Accessed February 18th 2025.

[11] Kouri TT Hofmann W Falbo R Oyaert M Schubert S Gertsen JB The EFLM European Urinalysis Guideline 2023. Clin Chem Lab Med. 2024;62:1653–786. 10.1515/cclm-2024-0070 38534005

